# Ivermectin Plasma Concentration in Iberian Ibex (*Capra pyrenaica*) Following Oral Administration: A Pilot Study

**DOI:** 10.3389/fvets.2022.830157

**Published:** 2022-04-01

**Authors:** Barbara Moroni, José Enrique Granados Torres, Jorge Ramón López-Olvera, José Espinosa Cerrato, Arián Ráez Bravo, Gregorio Mentaberre, Paulino Fandos, Marco Pazzi, Monica Romagnoli, Giulia Gardini, Luca Rossi, Marta Valldeperes, Emmanuel Serrano, Blanca Ramos, Rosangela Odore

**Affiliations:** ^1^Department of Veterinary Sciences, University of Turin, Turin, Italy; ^2^Parque Nacional y Parque Natural de Sierra Nevada Carretera Antigua Sierra Nevada, Granada, Spain; ^3^Wildlife Ecology & Health Group (WE&H) and Servei d'Ecopatologia de Fauna Salvatge (SEFaS), Departament de Medicina i Cirurgia Animals, Facultat de Veterinària, Universitat Autònoma de Barcelona, Barcelona, Spain; ^4^Departamento Sanidad Animal, Instituto de Ganadería de Montaña (CSIC-ULE), Facultad de Veterinaria, León, Spain; ^5^Wildlife Ecology & Health Group and Departament de Ciència Animal, Escola Tècnica Superior d'Enginyeria Agraria (ETSEA), Universitat de Lleida (UdL), Lleida, Spain; ^6^Agencia de Medio Ambiente y Agua, Sevilla, Spain; ^7^Department of Chemistry, University of Turin, Turin, Italy

**Keywords:** ivermectin, sarcoptic mange, wildlife, Iberian ibex, treatment, medicated feed

## Abstract

Sarcoptic mange is considered the main driver of demographic declines occurred in the last decades in Iberian ibex (*Capra pyrenaica*) populations. Mass treatment campaigns by administration of in-feed acaricides are used as a measure to mitigate the impact of mange in the affected populations. However, there are no data on ivermectin (IVM) pharmacokinetics in this wild caprine, and the treatment through medicated feed is not endorsed by evidence on its effectiveness. The aim of this study is to determine the pharmacokinetic profile of IVM in plasma samples of ibexes after the experimental oral administration of IVM, using high performance liquid chromatography (HPLC) with automated solid phase extraction and fluorescence detection. A dose of 500 μg of IVM per body weight was orally administered in a feed bolus to nine healthy adult ibexes (seven males and two females). Blood samples were collected by jugular venipuncture into heparin-coated tubes at day 1, 2, 3, 4, 7, 10, 15, and 45 post-administration (dpa). The highest plasma concentration of IVM (Cmax = 3.4 ng/ml) was detected 24 h after the oral administration (T1), followed by a rapid decrease during the first week post-administration. Our results reveal that plasma IVM concentration drops drastically within 5 days of ingestion, questioning the effectiveness of a single in-feed dose of this drug to control sarcoptic mange. To the best of our knowledge, this is the first study on plasma availability of oral IVM in ibexes and in any wild ungulate species.

## Introduction

Iberian ibex (*Capra pyrenaica*) is a wild ungulate native to the mountain ranges of the Iberian Peninsula and has recently been reintroduced in the French side of the Pyrenees. This caprine currently enjoys a favorable conservation status (“least concern” according to the IUCN), which allows sustainable hunting as a valuable game species. Besides its biological and ecological value, ibex trophy hunting and the associated related tourism represent a significant source of revenue for remote and disadvantaged rural communities in Spain (1). As for the majority of large herbivores in Europe, the hunting quotas are established by public conservation agencies, based on the estimated population size, although hunting is banned in specific protected areas such as National Parks (2, 3).

Since the late eighties of the twentieth century, free-ranging Iberian ibex populations suffered from epidemic waves of sarcoptic mange, a contagious skin disease caused by the burrowing mite *Sarcoptes scabiei*. After the epidemics, mange has remained endemic in all the populations affected. Devastating demographic effects have been recorded in naïve herds, including mortality rates over 90% (4, 5). Sarcoptic mange is now considered the main driver of short to medium term population declines in this wild ruminant (6).

Control of sarcoptic mange epizootics in wildlife is challenging. Amongst other measures like non–intervention (*laissez-faire*), massive lethal control, and selective culling of clinically-affected animals (7), individual and mass treatments with acaricides have been proposed and empirically implemented at local scale with unknown success (8). However, beyond ethical considerations, treating free-ranging wildlife has disadvantages, such as the impracticability of drug administration on a large population scale, and the potential environmental contamination with drug residues (9, 10). In Spain, the lack of shared protocols among regions to tackle the population decline caused by sarcoptic mange in exposed wild ruminants has promoted the empirical use of acaricides massively administered through medicated mixture feed (6). Nevertheless, no studies support the actual effectiveness of this common practice. In fact, essential gaps in knowledge need to be filled before considering oral mass treatments as a possible control measure for the management of sarcoptic mange in free-ranging Iberian ibex and other susceptible wild ruminants (11, 12). Among the knowledge gaps to fill before implementing any in-field treatment, the pharmacokinetics of orally-administered candidate acaricides in the target hosts are crucial. Pharmacokinetic is the keystone to establish effective drugs, dosages, vehicles, frequency of administration, number of feeding points according to surface area and population, and uptake rates of medicated feed that should be reached in order to obtain a mass effect limiting the impact of the disease under field conditions. Difficulties in recruiting, maintaining and repeatedly handling Iberian ibexes have, so far, understandably limited the necessary trials.

In captive wild ruminants, the macrocyclic lactone ivermectin (IVM) has been used against *S. scabiei* infection at dose rates of 200–400 μg/kg, and repeated subcutaneous administrations were necessary to eliminate severe clinical signs (8). Oral administration has also been described in wild free-ranging animals (6, 8, 13); nevertheless, the plasma concentration in treated individuals, and the drug plasma therapeutic concentrations against sarcoptic mange were unknown, as well as the proportion of individuals having access to medicated feed. In domestic ruminants, interspecies and intra-species variability in IVM pharmacokinetics has been observed. In domestic goat, a greater plasma clearance of IVM leads to lower plasma concentrations than in cattle and sheep, regardless of the route of drug administration (14). After oral administration, IVM bioavailability is about four times greater and plasma concentrations can be detected longer in sheep than in goats (15). Moreover, IVM pharmacokinetics may depend on sex and age, and the presence of rumen digesta significantly influences the systemic availability of the drug (16).

The main findings related to domestic ruminants and the paucity of data concerning wild species recommend caution when extrapolating treatment protocols (e.g., dose and formulation) from one species to another and, as a consequence, extended pharmacokinetic data in target animal species are much-needed to avoid drug misuse. Therefore, the aim of the present experimental study is to begin filling the existing knowledge gaps by exploring, for the first time, the pharmacokinetic profile of a single oral dose of IVM in Iberian ibexes.

## Materials and Methods

### Animals

Nine adult healthy Iberian ibexes (seven males and two females, age ranging 1–5 years) were captured in Sierra Nevada Natural Space as part of the regular management of the species and transferred to the “Iberian ibex stock reservoir El Toril”, in Dílar, Granada (37°02′-7°03′N, 3°22′-3°33′W), southern Spain (17). The ibexes were kept isolated from the rest of the reservoir ibex population during all the study period. Average weight was 19 kg for females and 26 kg for males. The study was approved by the Ethics on Animal Welfare Committee of the University of Jaén and authorized by the Dirección General de Producción Agrícola y Ganadera of the Consejería de Agricultura, Pesca y Medio Ambiente of the Junta de Andalucía (Ref: SA/SIS/MD/ps/October 25, 2012). The Sierra Nevada National and Natural Park Administration also approved this study.

### Pharmacotherapy and Blood Sample Collection

After an acclimation period of at least 1 month in the experimental facilities, an *ad hoc* formulated bolus containing 0.5 mg of IVM per kg of body weight was orally administered to each ibex. For this, specialized wildlife operators physically immobilized, blindfolded, and administered the boluses to the ibexes using commercial applicators. Blood samples (20 ml) were collected from the jugular vein into heparin-coated vacutainers just prior to drug administration (time 0) and 1 (T1), 2 (T2), 3 (T3), 4 (T4), 7 (T5), 10 (T6), 15 (T7), and 45 (T8) days after the bolus administration (dpa). Plasma was obtained immediately after the blood collection by centrifugation at 2,000 g for 20 mins and stored in 2 ml containers at −80°C until analyzed.

### Chemical Extraction, Derivatization and HPLC With Fluorescence Detection

IVM was analyzed in the plasma samples by high performance liquid chromatography (HPLC) with automated solid phase extraction and fluorescence detection following a validated method previously described (18). Briefly, 1 ml plasma aliquot was mixed with 5 ng internal standard abamectin and vortexed. The solution was mixed with 1 ml acetonitrile/water solution (4:1) for 30 mins and then centrifuged at 17,000 g at room temperature for 5 mins. The resultant supernatant was applied at 10 mg/ml to a cartridge (Strata-X 33 μm Polymeric Reversed Phase, Phenomenex, Torrance, CA, US), previously activated with 1 ml methanol and 1 ml of water. Elution was performed with 1.3 ml methanol. The dried residue was dissolved in 100 μl N-methylmidazole solution in acetonitrile (1:2) and derivatized with 150 μl trifluoroacetic anhydride solution in acetonitrile (1:3).

A 100-μl aliquot of the derivatized samples was injected into the chromatograph. The mobile phase consisted of acetic acid 0.2% in water/methanol/acetonitrile (10:40:50) pumped at a flow rate of 1.4 ml/min through a column (Gemini C18, 150 x 4.6 mm, Phenomenex) with fluorescence detection. Fluorescence detection (RF 2000 Fluorescence Detector, Dionex) was performed at 365 nm excitation and 475 nm emission wavelength. Recovery rate was calculated for each sample through internal standard recovery correction.

### Data Analysis

A Linear Mixed Model (LMM with a normal error distribution and identity link function) was fitted to explore the pharmacokinetics of plasma IVM concentrations (ng/ml, log-transformed) in the study ibexes. Time since drug administration was included as fixed factors whereas ibex identity as a random intercept term in the LMM (19). For the mixed models we used the library lme4 1.1-15 version (19), whereas the library MuMIn 1.43.6 version (20) was employed to assess the marginal and conditional contribution of the fixed and random terms (21). All the statistical analysis were performed with R Statistical Software 4.1.0 version (22).

## Results

**Figure 1:**
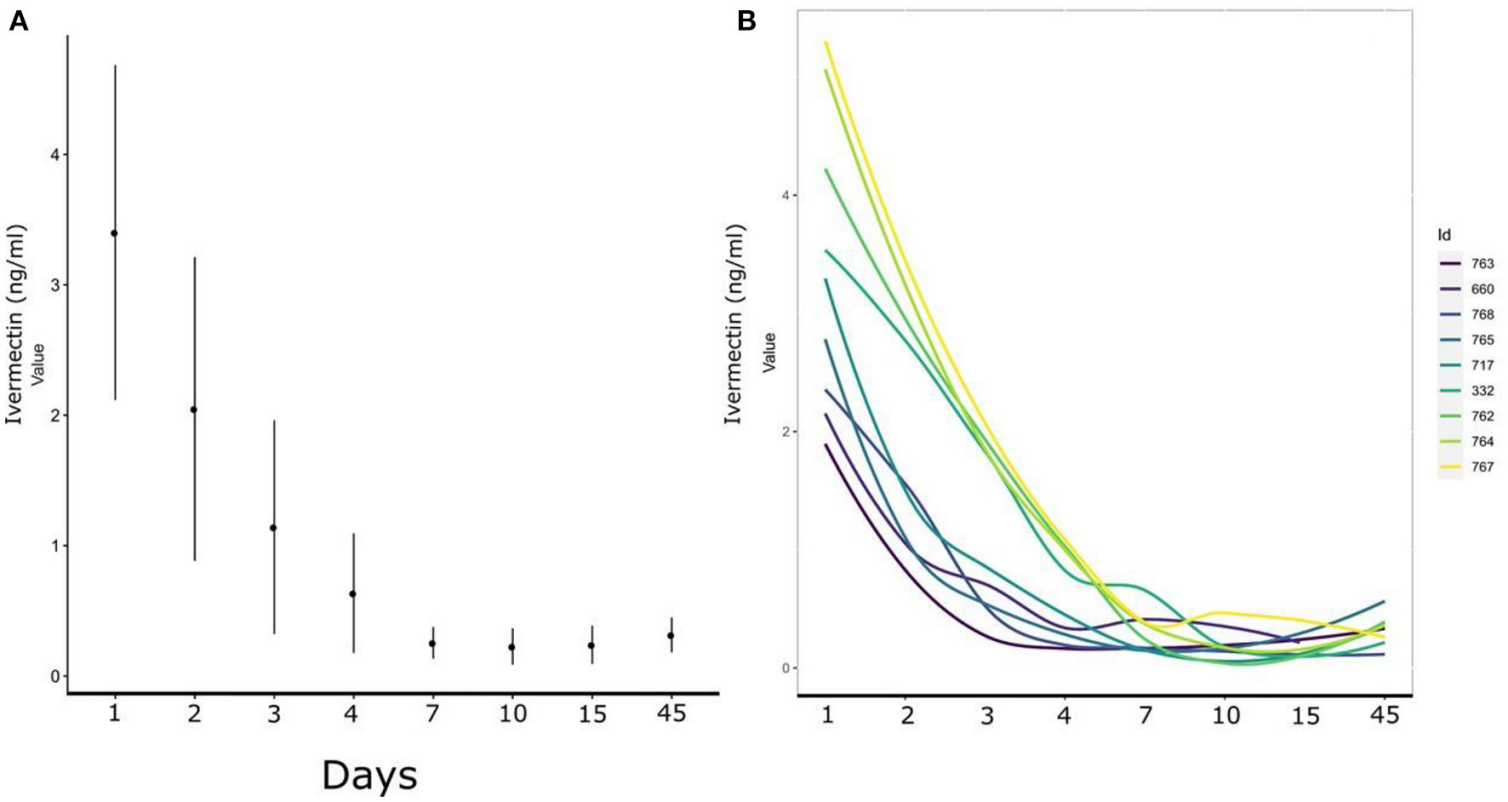
**(A)** Time trend of the mean plasma ivermectin concentration (ng/ml) in the nine Iberian ibexes orally administered with ivermectin from 1 (T1) to 45 (T8) days post-administration; **(B)** Differences among individual ibexes in the decrease of plasma ivermectin concentration throughout the study period.

The limit of quantification was 0.2 ng/ml. Although IVM was detected in plasma from dpa 1 to dpa 45, mean plasma IVM concentration dropped from the initial 3.40 ng/ml in dpa 1 to 0.63 ng/ml in dpa 4 (Figure 1A), and stabilized thereafter around 0.25 ng/ml. This temporal decrease (β = −0.4, SE = 0.04, *t* = −11.11, *p*-value < 0.01) explained 61% of the plasma IVM concentration variability in the ibexes of the study. Moreover, the individual contribution to the observed patterns was 8% (Figure 1B).

The pharmacokinetic values of orally-administered IVM in the Iberian ibexes are reported in Table 1. No adverse response was observed throughout the study.

**Table 1:** Ivermectin pharmacokinetic values in plasma of the experimental Iberian ibexes after the oral administration of a 0.5 mg/kg dose.

**Cmax (ng/ml)**	**Tmax (days)**	**AUC (days ·ng/ml)**	**Tlast (day)**	**MRT (days)**
3.40 ± 1.2	1	21.27	45	30.80

*Cmax, maximum concentration; Tmax, time for maximum concentration; AUC, Area Under Curve; Tlast, time of last detection of ivermectin; MRT, mean residence time*.

## Discussion

To the best of our knowledge, this is the first study investigating the pharmacokinetic profile of orally administered IVM in Iberian ibex and in any wild ruminant species. The controlled conditions of the experiment will allow the use of the results as reference for future experimental scenarios and/or field studies. Sarcoptic mange has been pharmacologically treated in captive wildlife, and some experiences in free-ranging wildlife populations have also been carried out, mostly empirically (8, 10, 23).

However, the complete eradication of mange from a population in endemic areas through pharmacological control strategies has been never achieved and so far deemed as unrealistic or inconclusive (10).

Literature is poorly informative on the efficacy of different doses of orally administered macrocyclic lactones for the treatment of sarcoptic mange in wild ruminants (8). Hence, the IVM dose in this study (0.5 mg/kg) must be intended as an empiric trade-off between a limited number of relevant sources. Amongst them, Yeruham and colleagues (24) successfully treated five ruminant species affected by sarcoptic mange in zoo gardens, including the Nubian ibex (*Capra nubiana*), a close relative of the Iberian ibex. Their protocol provided for the administration of an oral dose of 0.2 mg/kg of IVM for three consecutive days repeated three times at 2 week intervals. This protocol is clearly unfit for mass use under free-ranging conditions, since regular access of target wildlife to medicated feed is far from being guaranteed; nevertheless, the results are encouraging with respect to possible oral treatment options in *Sarcoptes*-exposed wild ruminants. Moreover, Leon-Vizcaino and colleagues (25) showed that 0.4 mg/kg of subcutaneously administered IVM were preferable to 0.2 mg/kg for the treatment of spontaneously infested Iberian ibex, and Foreyt (26) reported the successful in-feed use of IVM for 7 consecutive days at the dose of 1 mg/kg for the treatment of Rocky Mountain bighorns (*Ovis canadensis*) experimentally infested with *Psoroptes* mites. The 0.5 mg/kg IVM dose in our study took into account the above findings, the lower antiparasitic efficacy of oral IVM when administered at the same dosage recommended via subcutaneous inoculation (27) and, prospectively, the individual variability in medicated food intake by free-ranging wild ruminants.

This study revealed that IVM plasma concentrations in orally-treated Iberian ibexes drop drastically within four dpa. Not surprisingly, the oral route leads to lower IVM plasma concentrations compared to those reached after a subcutaneous administration (28), likely due to the binding to the particulate phase of digesta (14, 29). Moreover, the binding of IVM to plasma albumin and lipoproteins should be taken into account especially in animals with chronic stages of sarcoptic mange usually showing emaciation and poor body condition, in which a decrease in plasma proteins (thus a higher free fraction of IVM in the plasma), might be more likely associated to an unsuccessful treatment (14).

The maximum drug concentration and bioavailability found in Iberian ibex (3.40 ± ng/ml) were lower than those described in goats (6.03 ± 0.95; 15.85 ± 5.29 ng/ml, respectively) after the oral administration of an IVM dose of 0.2 mg/kg (30, 31). By contrast, the time to maximum concentration (1 day) was consistent with previous reports of oral administration in other medium-sized ruminants, sheep (1.7 day), goat (2.8 days), and reindeer (2 days for oral mixture, 1 day for oral paste) (15, 18, 28). By comparing two different oral formulations in reindeer, Oksanen et al. (28) found a lower relative plasma availability for a paste than for an oral liquid drench formulation, which could also partially explain the lower values observed in this study. The solid bolus formulation used in this study does not appear in previous similar trials, but we deemed it: i) better representative, than a liquid drench, of the medicated pellet administration that is already empirically used in Iberian ibex (32); ii) better suited to provide an accurate and precise IVM dosing than medicated pellets.

Several factors known to influence drug absorption and bioavailability may explain the interindividual variability in IVM plasma concentrations, observed in particular at T1-T4 (33). Nevertheless, as anticipated, the variability could be even greater under field conditions (e.g., mass-delivery treatment schemes with delivery of medicated feed), thus the variability in the IVM plasma concentrations reached should likely be greater due to inter-individual variability in medicated food intake, with unpredictable consequences on IVM efficacy. The antiparasitic action of macrocyclic lactones is related to their plasma concentrations, and more specifically to the bioavailability (34). On the other hand, due to its high lipophilic nature, IVM is widely distributed within the body in all species, with highest concentrations achieved in liver and adipose tissue, where it tends to accumulate (14). This might explain the rapid drop of IVM observed in the plasma of Iberian ibexes in this study, as well as the long MRT (30.8), pointing to a possible quick deposit in other tissues. Interestingly, it has been experimentally observed that IVM is detectable in the skin of scabietic pigs for at least 10 days after a single oral administration of 0.2 mg/kg (35).

Therapeutic or effective threshold for IVM plasma concentrations against sarcoptic mange has not been defined in any ruminant model. In cattle, the minimum IVM blood concentration for anthelmintic activity against nematodes ranges from 0.5 to 1 ng/ml (36). By contrast, a higher therapeutic threshold level (8 ng/ml) has been suggested after a subcutaneous injection of 0.6 mg/kg long-acting IVM against common bovine ticks (*Boophilus spp*) (37), showing that therapeutic blood levels may depend, among others, on the animal species and related metabolism, as well as the target parasite. Moreover, the therapeutic efficacy and the associated threshold of IVM plasma concentrations may vary depending on the severity of sarcoptic mange, with the most affected individuals being less responsive to the therapeutic effect, thus requiring a higher dosage to eliminate the mite and heal (30), as shown in other animal species (31).

The efficacy of IVM plasma concentrations against *S. scabiei* in Iberian ibex and other wild ungulate species should be further investigated, also considering that underdosing is a recognized driver for the development of resistance to endo and ectoparasiticides (8, 10, 38, 39). At present, resistance of *S. scabiei* against IVM has been described both, *in vitro* and *in vivo*, limited to human model (40, 41). In the particular case of sustained oral administration of IVM to free-ranging Iberian ibexes, resistance to the drug could be also developed by generalist nematodes of the digestive tract, eg the blood-feeding *Haemonchus contortus*, that may be cross-transmitted with sympatric sheep and goats (32, 42). This could result in negative herd health management issues at the wildlife/livestock interface (23).

Some limitations of the present study include the relatively low number of dosed individuals and the absence of time points within the first 24 h post treatment, which would have provided more precise information concerning IVM absorption rate and plasma maximum concentrations.

The pharmacological treatment of sarcoptic mange in wildlife is a complex and controversial management measure, with absence or paucity of protocols regarding dosage, administration times, density of medicated baits per surface or animal population unit, and a lack of knowledge on the pharmacokinetics of macrocyclic lactones in most wildlife species, on the chances for resistance to appear due to underdosage and on the potential environmental consequences of the massive release of antiparasitic drugs in the environment (8, 10, 43–45). In Iberian ibex, traditional management has mostly relied in decreasing host density and/or the selective culling of affected individuals (7, 46). However, short-term strategies including the non-selective mass administration of medicated feed to affected ibex populations has also been tried, with little or no effect on the disease prevalence and demographic impact (6, 47, 48). The rapid drop of plasma IVM concentration in Iberian ibex after a single oral dose found in this study, and the lack of published results on therapeutic trials with oral IVM in scabietic free-ranging individuals suggest caution in promoting the mass delivery of IVM medicated pellets as a measure to efficiently control sarcoptic mange at the individual and population level in this wild caprine. The epidemiological effects of partially ineffective treatment strategies could even be opposite, by lengthening the infective stage of the individuals affected by sarcoptic mange and therefore increasing the dissemination and spread of the disease (25), potentially to other mammal species too (12, 49). Moreover, aspects such as the proportion of the target population actually receiving the treatment, the access to the drug by non-target species, and the environmental effects of the massive release of IVM in the environment, are still in need to be thoroughly investigated and controlled (8, 10). A major concern is that IVM and its metabolites are mainly eliminated in the feces of treated individuals (50), with potential impact of the residues on the biology and reproduction of dung invertebrate fauna (45, 50).

*In vitro* and *in vivo* studies carried out with *Sarcoptes* mites var. *suis* showed that moxidectin concentration required to kill 50% of mites was lower than that of IVM, and that a single dose of moxidectin or fluralaner were more effective than two consecutive of IVM, suggesting that IVM should be soon replaced in the treatment approach for sarcoptic mange (35, 51, 52). New long-acting oral and topic isoxazolines are emerging as more efficient therapeutic options against sarcoptic mange, as suggested by recent experimental studies in both domestic animals and captive wildlife (52–54).

Nonetheless, and similarly to IVM, numerous gaps in knowledge must be addressed before the in-field use of these alternative drugs may be considered a valid option (10). Recently, Mounsey et al. (55) highlighted the precarious balance between dosage, route of administration, ecotoxicity, drug resistance and efficacy of macrocyclic lactones in the Australian wombats (*Vombatus ursinus* and *Lasiorhinus latifrons*), two highly susceptible wildlife species to sarcoptic mange, suggesting that a better cooperation and a continuous debate between stakeholders, including veterinarians, wildlife carers and researchers, should be encouraged to achieve best treatment options for wildlife.

There is therefore a need to further investigate the pharmacokinetics, pharmacodynamics, population target and environmental consequences of the administration of available macrocyclic lactones, isoxazolines and other candidate molecules, including long-acting formulations, before their in-field delivery become a management option to control sarcoptic mange in free-ranging wild ruminants.

## Data Availability Statement

The original contributions presented in the study are included in the article/supplementary material, further inquiries can be directed to the corresponding author/s.

## Ethics Statement

This study complied with all Andalusian, Spanish, and European legal requirements and guidelines regarding experimentation and animal welfare. Handling procedures and sampling frequency were designed to reduce stress and minimize the impact on the health of the subjects, as per European (2010/63/UE) and Spanish (R.D 53/2013) standards. The study was approved by the Ethics on Animal Welfare Committee of the University of Jaén and authorized by the Dirección General de Producción Agrícola y Ganadera of the Consejería de Agricultura, Pesca y Medio Ambiente of the Junta de Andalucía (Ref: SA/SIS/MD/ps/ October 25, 2012). The Sierra Nevada Natural Park staff also approved this study.

## Author Contributions

BM, JG, JL-O, JE, AR, GM, PF, LR, MV, ES, BR, and RO conceptualized the project and agreed on the study design. RO prepared the IVM bolus. JG, JE, AR, and BR performed the IVM treatment and obtained blood samples from the ibexes. BM, GG, MP, and MR performed the laboratory analysis and elaborated the results. ES performed the statistical analysis. BM drafted the original manuscript. JG, JL-O, JE, AR, GM, PF, LR, MV, ES, BR, and RO contributed in shaping and revising the manuscript. All the authors have read and agreed to the final version of the manuscript.

## Funding

This project was funded by the Consejería de Medio Ambiente de la Junta de Andalucía (project 173/2009/M/00; 03/15/M/00; 861_11_M_00 and 2016/00014/M), and by the Spanish Ministerio de Economía y Competitividad (projects CGL2012-40043-C02-01, CGL2012-40043-C02-02, and CGL2016-80543-P). The authors' research activities are partially supported by the Plan Andaluz de Investigación (RNM-118 group). MV is supported by a FI-GENCAT Fellowship (2020_FI_B2_00049, co-financiated by Agència de Gestió d'Ajuts Universitaris i de Recerca and European Social Fund) and ES by the Spanish Ministerio de Ciencia Innovación y Universidades (MICINN) through a Ramon y Cajal agreement (RYC-2016-21120). GM is a Serra Húnter Fellow.

## Conflict of Interest

The authors declare that the research was conducted in the absence of any commercial or financial relationships that could be construed as a potential conflict of interest.

## Publisher's Note

All claims expressed in this article are solely those of the authors and do not necessarily represent those of their affiliated organizations, or those of the publisher, the editors and the reviewers. Any product that may be evaluated in this article, or claim that may be made by its manufacturer, is not guaranteed or endorsed by the publisher.

## Abbreviations

IVM: ivermectin
HPLC: high pressure liquid chromatography.
